# EmptyDropsMultiome discriminates real cells from background in single-cell multiomics assays

**DOI:** 10.1186/s13059-024-03259-x

**Published:** 2024-05-13

**Authors:** Stathis Megas, Valentina Lorenzi, John C. Marioni

**Affiliations:** 1 European Molecular Biology Laboratory European Bioinformatics Institute, Hinxton, Cambridge, UK; 2 Wellcome Sanger Institute, Wellcome Genome Campus, Hinxton, Cambridge, UK; 3 Cancer Research UK Cambridge Institute, University of Cambridge, Cambridge, UK; 4 Wellcome Trust/CRUK Gurdon Institute, Department of Physiology, Development of Genetics, University of Cambridge, Cambridge, UK

**Keywords:** Multiomics, Single-cell, Method

## Abstract

Multiomic droplet-based technologies allow different molecular modalities, such as chromatin accessibility and gene expression (scATAC-seq and scRNA-seq), to be probed in the same nucleus. We develop EmptyDropsMultiome, an approach that distinguishes true nuclei-containing droplets from background. Using simulations, we show that EmptyDropsMultiome has higher statistical power and accuracy than existing approaches, including CellRanger-arc and EmptyDrops. On real datasets, we observe that CellRanger-arc misses more than half of the nuclei identified by EmptyDropsMultiome and, moreover, is biased against certain cell types, some of which have a retrieval rate lower than 20%.

**Supplementary Information:**

The online version contains supplementary material available at 10.1186/s13059-024-03259-x.

## Background

Droplet-based assays have enabled efficient and high-throughput measurement of multiple molecular modalities at single-cell resolution, including single-cell gene expression (GEX) profiling (single-cell RNA-sequencing (scRNA-seq)) [1, 2] and single-cell chromatin accessibility (Assay for Transposase-Accessible Chromatin; ATAC) profiling (scATAC-seq) [3, 4]. Moreover, recent advances have made it possible to assay both molecular modalities in the same single-cell (or technically, the same nucleus) enabling study of the coupling between distinct molecular layers [5–7]. The joint profiling of RNA and chromatin accessibility enables direct matching of transcriptional regulation to its output, achieving a more complete reconstruction of cellular processes at the molecular level. In a typical single-cell multiomic (sc-multiomic) ATAC + GEX experiment, nuclei suspensions are incubated alongside a transposase. Subsequently, nuclei are loaded into droplets that contain (1) oligonucleotides with a spacer sequence that enables barcode attachment to transposed DNA fragments, (2) primers with a poly-(dT) sequence to uniquely barcode poly-adenylated mRNA molecules, and (3) reagents to reverse-transcribe the barcoded mRNA molecules into cDNA. Barcoded DNA and cDNA are then PCR amplified and used to construct ATAC and GEX libraries. After sequencing, reads from each library can be uniquely associated with the originating nucleus thanks to these added barcodes.

While loading the nuclei into droplets, some of the buffer in which they are suspended is also captured. Moreover, some droplets fail to capture any nuclei—and therefore only contain the buffer. In an ideal setting, the buffer in which the nuclei are suspended is completely devoid of RNA or DNA molecules, meaning that each droplet either has no reads or only reads originating from a single nucleus. In practice, however, the entrance of the transposase requires permeabilization of the nuclei, which causes fragments of DNA and RNA to escape, meaning that the buffer is a soup of RNA and DNA molecules from different cells [1]. As a result, nuclei-free droplets can exhibit non-zero read counts, potentially leading them to resemble true nuclei and thereby confounding downstream analysis.

To the best of our knowledge, there is currently only one method for detecting nuclei-containing droplets in sc-multiome ATAC + GEX data. This forms part of the CellRanger-arc software developed by 10x  Genomics and is tailored to its experimental protocol [8]. Nuclei detection is performed by CellRanger-arc in two steps: (1) filtering and (2) nuclei calling (which they refer to as cell calling).

### Removing nuclei using the fraction of reads in peaks (FRiP)

In the filtering step, the CellRanger-arc software first identifies peaks, i.e., consensus regions of the genome which are present in the majority of cells (“bulk peaks”, padded by 2000 bp on each side). Subsequently, it tests, for each cell, whether the ATAC-seq fragments are concentrated within these bulk peaks. If not, the droplet is considered empty and removed. While this filtering approach works well for homogeneous samples, where all cells are expected to share the same “bulk peak” calls, it might be problematic for heterogeneous samples containing different cell types. In such cases, nuclei belonging to low abundance cell types or those possessing distinct chromatin accessibility patterns might be erroneously removed during filtering. Finally, these problems are more pronounced in species with smaller genomes since the (uncustomizable) padding of the peaks by 2000 bp on each side increases the fraction of genome in peaks, which in turn sets a higher bound on the FRiP of nuclei that will be accepted as valid during the filtering process.

To illustrate the problems of adopting this filtering strategy, we consider real single-nucleus multiome datasets from a recent study on human gonadal development. In [9], the authors generated a comprehensive map of first- and second-trimester human gonads using a combination of single-cell and spatial genomics assays, including 10x multiome. Their analyses helped elucidate the mechanisms underlying the differentiation of primordial germ cells (PGCs) into either pre-spermatogonia or oocytes via signaling from the surrounding somatic cells, which themselves undergo differentiation in two stages from coelomic epithelial to supporting to pregranulosa cells. To illustrate the challenges of using the default 10x approach for filtering and calling cells, we focused on two samples from a single female donor at 21 weeks post-conception (hereafter sample A and sample B) [10].

**Fig. 1:**
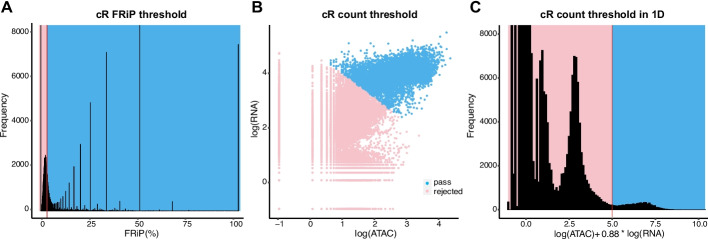
Evaluating the performance of CellRanger-arc (cR). **A** Histogram of the fraction of reads in peaks (FRiP) of all droplets for sample A. Nuclei containing droplets are located within the bell curve and in the right tail of the distribution. The CellRanger-arc threshold (vertical red line) rejects more than half of the nuclei within the bell curve. **B** CellRanger-arc count threshold. A scatter plot shows the number of log(ATAC) reads (*x*-axis) versus the unique log(RNA) reads (*y*-axis) for each droplet, and we observe a continuum in the distribution of counts. Nuclei that pass CellRanger-arc’s count threshold are plotted in blue, and those that fail this threshold are plotted in pink. **C** Histogram of a linear combination of logarithmic counts that is constant along the k-means line. We see a continuum at the location of the threshold (vertical red line)

As shown in Fig. 1A, the FRiP threshold inferred by CellRanger-arc for sample A is larger than the mode of the distribution. This contrasts with typical quality control recommendations for scATAC-seq data, which suggest discarding droplets where the FRiP is less than 3 median absolute deviations (MADs) below the median FRiP [11]. We speculated that the conservative CellRanger-arc threshold was driven by inclusion of spurious genomic peaks and the 2 kb padding, which increases the fraction of the genome that falls in peaks. Supporting our hypothesis, excluding the shortest 10% of “bulk” ATAC peaks, which are most likely to be erroneous, yields a FRiP threshold that is 0.002 smaller, leading to the inclusion of an additional 5000 droplets.

### Removing nuclei using ATAC and RNA counts

Following application of this initial filter, CellRanger-arc next uses the number of ATAC and RNA counts to generate a secondary filter. Specifically, droplets are clustered into nuclei-containing and nuclei-free populations using k-means (*k* = 2) clustering in the space of log transformed ATAC and RNA counts (see Fig. 1B, C). K-means clustering is a hard cut-off classifier, which is problematic in cases where the buffer has high ambient noise (i.e., contains many RNA and ATAC fragments that were released upon permeabilization of the nuclei). In particular, this can lead to a continuous distribution of counts, with no clear separation between the population of nuclei-containing droplets and that of nuclei-free droplets (see Fig. 1B, C). Consequently, similar to the first filtering step employed by CellRanger-arc, this can result in the inadvertent removal of nuclei with few accessible regions and/or transcripts expressed.

More mathematically rigorous models exist for detecting cell-free droplets in scRNA-seq experiments [12–14]. The key concept behind the popular EmptyDrops approach [12] (later also incorporated into the scRNA CellRanger pipeline [15]) is to profile the soup, i.e., the noise in the dataset, before testing whether the distribution of gene expression counts in putative cells deviates from this profile. This approach is agnostic to cell types and avoids the homogeneity assumptions of CellRanger-arc. For cell calling on multiome data, EmptyDrops on the RNA modality could be combined with a hard cut-off threshold on the ATAC library size. However, for the reasons outlined above, and as exemplified in the original EmptyDrops study, any hard cut-off method will likely be sub-optimal, especially for cell types with distinct and sparse chromatin accessibility profiles.

Here, we propose a new method for discerning nuclei-containing droplets in droplet-based sc-multiomics data. By generalizing EmptyDrops [12, 16] to the multiomic setting, we use the smallest droplets to create the RNA profile and the ATAC profile of the soup, and then test each droplet for statistical deviations from each of these two profiles, retaining droplets that are statistically significantly different from the soup. We combine this with an optional hard cut-off filter to ensure that barcodes with very large total counts are always retained. Using simulations, we demonstrate that our approach outperforms both any method based on library-size thresholds (like CellRanger-arc) and EmptyDrops. Additionally, we show that by applying our method to real datasets we can recover more cells, including those from cell types that are almost entirely discarded by CellRanger-arc.

## Results

### Evaluating performance on simulations based on real droplet-based data

We evaluated the performance of EmptyDropsMultiome using simulations based upon a high-quality multiome (ATAC + GEX) dataset generated from peripheral blood mononuclear cells (PBMCs) and published by 10x [17]. This sample is from a female donor aged 25 and was profiled following removal of granulocytes by cell sorting. Granulocyte sorting is recommended by 10x for samples with high granulocyte content since, during NETosis, their chromatin becomes very accessible which can both decrease the sequencing depth of other cell types as well as causing CellRanger-arc to misclassify granulocytes as dead cells [18]. The simulations based on the PBMC dataset were designed to test the ability of CellRanger-arc and EmptyDropsMultiome to correctly call nuclei from cell types with little chromatin accessibility and total overall expression.

**Fig. 2:**
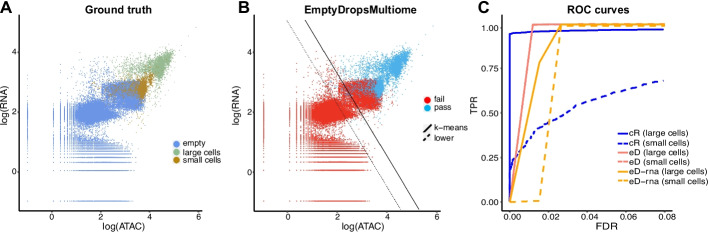
Simulations to assess the performance of different cell calling approaches. **A** Scatterplot showing the total number of RNA and ATAC counts for the 5000/2000 simulated dataset with one new cell type. Ground truth of the simulated dataset: all the empty droplets from the PBMC dataset and additional simulated empty droplets, 5000 nuclei containing droplets, 2000 small simulated nuclei by subsampling RNA + ATAC and then scrambling the genomic profile of monocytes. **B** Result of applying EmptyDropsMultiome at FDR = 0.1% on the 5000/2000 simulation. The nuclei containing droplets are singled out from among the empty ones. The solid line shown is the k-means line used by CellRanger-arc, which misses all the points below it. **C** ROC curve comparison on the 5000/2000 simulation of EmptyDropsMultiome against a customizable version of CellRanger-arc where we change the intercept of the k-means line (while maintaining its slope)

To begin our simulation, we assume that CellRanger-arc has correctly identified nuclei-containing and nuclei-free droplets. Subsequently, we generated simulated datasets as follows (see Fig. 2A):

- We sample ${g}_{1}$ (CellRanger-arc identified) nuclei.
- We perform clustering on the population of CellRanger-arc identified nuclei, and then we sample ${g}_{2}=2000$ nuclei from the CD14 + cluster of cells, which we identify as monocytes. For each of these monocytes, we randomly sample 20% of the RNA reads and 20% of the ATAC reads. Additionally, we randomly shuffle the identity of 10% of the expressed genes and 10% of the region names. This is supposed to simulate a new cell type (because of the scrambling) with low levels of transcription and limited genome accessibility (due to the downsampling or reads).
- We retain all of the CellRanger-arc identified empty droplets.
- We simulate more empty droplets to create a continuum in the space of counts. We do this by taking droplets in the ambient cluster and multiplying their count vector by random integers.
- The ${g}_{1}+{g}_{2}$ cells and the empty droplets constitute our simulated dataset.

We generate simulations for ${g}_{1} \epsilon \{2000, 5000\}$ to consider situations where the altered cell types ${g}_{2}$ are present at different relative frequencies. Finally, we apply EmptyDrops and EmptyDropsMultiome (both at an FDR threshold of 0.1%) as well as CellRanger-arc to the simulated datasets. All three methods find the populations of small and large cells but only EmptyDropsMultiome does so without yielding a large number of false positives (Fig. 2B and Additional file 1: Fig. S4).

In addition to using two hard cut-offs for cell calling, another limitation of CellRanger-arc is that the user cannot customize how conservative those cut-offs are. To assess how these hard cutoffs impacted the method’s performance, we implemented a customizable version of CellRanger-arc that allows the threshold to be altered by shifting the k-means line of CellRanger-arc parallel to itself. To assess the impact of using different k-means thresholds, as well as the choice of the False Discovery Rate (FDR) threshold, we used receiver operating characteristic (ROC) curves to compare the performance of EmptyDrops, EmptyDropsMultiome, and CellRanger-arc.

As can be seen in Fig. 2C, at a given FDR threshold, EmptyDropsMultiome outperforms both EmptyDrops and CellRanger-arc. EmptyDropsMultiome’s systematic outperformance of EmptyDrops shows that neglecting the ATAC modality leads to a larger fraction of false positive calls being made. Moreover, when focusing on the simulated population with low levels of transcription and chromatin accessibility, the threshold-based approach implemented by CellRanger-arc performed more poorly than both EmptyDrops and EmptyDropsMultiome. This suggests that CellRanger-arc performs sub-optimally for cell calling in samples where the spectrum of counts varies continuously from empty to nuclei-containing droplets.

Moreover, to demonstrate that the results of the simulations do not depend on the choice of monocytes as the basis for the simulated new cell types, we also perform simulations where the new cell types generated as part of the simulation are created by downsampling and scrambling any CellRanger-arc identified cell population. The results of these simulations are consistent with those presented above (see Additional file 1: Fig. S5 and Methods). Finally, to show that our findings are not dependent upon the way we add noise to our simulated empty droplets, we performed additional simulations where a low signal-to-noise is achieved by downsampling 2000 real cells down to noise levels (6% of RNA and 2% of ATAC), without simultaneously upsampling the noise. These simulations reproduce our superior performance against competing methods and in fact showcase how CellRanger-arc can miss small cell types that lie under the k-means line (see Additional file 1: Fig. S8).

### Evaluating performance on real data

**Fig. 3:**
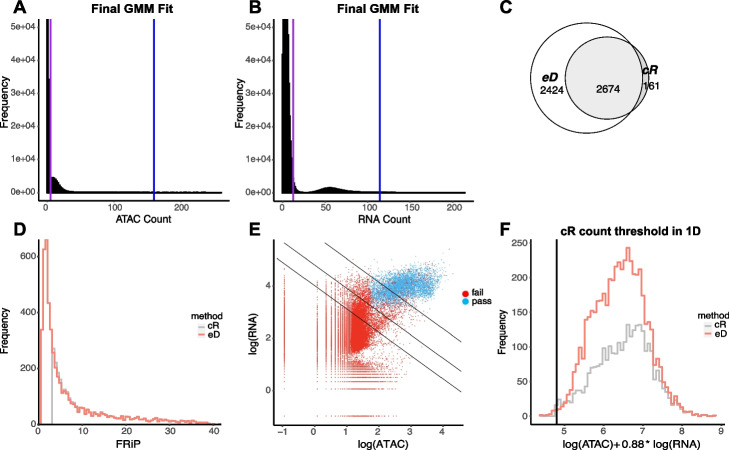
Results of the application of EmptyDropsMultiome on sample A after quality control. **A**, **B** Histograms of ATAC and RNA counts of all the droplets. We profile the soup using only the ambient cluster in ATAC and RNA. The lower and upper bound of the cluster are marked by the purple and blue line and are deduced via a Gaussian mixture model. **C** Venn diagram showcasing that the droplets selected by CellRanger-arc (cR) are almost a subset of those selected by EmptyDropsMultiome (eD). **D** Histogram of the FRiP for all droplets selected by CellRanger-arc or EmptyDropsMultiome after quality controls. A very large number of droplets retained by EmptyDropsMultiome has FRiP below the threshold imposed by CellRanger-arc. **E** The distribution of droplets selected by EmptyDropsMultiome in the space of logarithmic counts. A very small number of droplets selected by EmptyDropsMultiome lies below the k-means line (middle line shown) and is likewise excluded due to CellRanger-arc’s hard cut-off on library size. For upper and lower lines, see the “Methods” section. **F** The histogram of the linear combination of logarithmic ATAC and RNA counts that is constant along the k-means line. Again, we see a small number of droplets that CellRanger-arc misses due to this hard cut-off

Having assessed its performance on simulated data, we next applied EmptyDropsMultiome to the human gonadal development datasets introduced earlier. As seen in Fig. 3A, B, EmptyDropsMultiome identifies groups of cells that likely correspond to the ambient clusters in both the RNA and ATAC modalities. When applying the statistical test to identify nuclei-containing droplets, we note that EmptyDropsMultiome finds roughly twice as many droplets as CellRanger-arc, even after application of stringent quality control thresholds (see Fig. 3C and the”Methods” section).

As expected, the nuclei called exclusively by EmptyDropsMultiome correspond to those with either a small library size or with a small fraction of fragments in peaks. Indeed, in each of samples A and B, EmptyDropsMultiome retains thousands more cells below the arbitrary FRiP threshold CellRanger-arc imposes (Figs. 3D and S6). It also retains 28 nuclei (39 in sample B) with library size smaller than the k-means threshold used by CellRanger-arc (Fig. 3E, F).

Of note, before applying any explicit quality control threshold on the FRiP, the distribution of FRiP for nuclei identified by EmptyDropsMultiome was bounded away from 0 (Fig. 3D), and only 8 of the called droplets (7 in sample B) have FRiP less than 1 MAD below the median. Moreover, the spurious discrete peaks in FRiP of Fig. 1A have similarly been eliminated. These observations suggest that application of EmptyDropsMultiome acts as an appropriate and cluster-free quality control for ATAC.

**Fig. 4:**
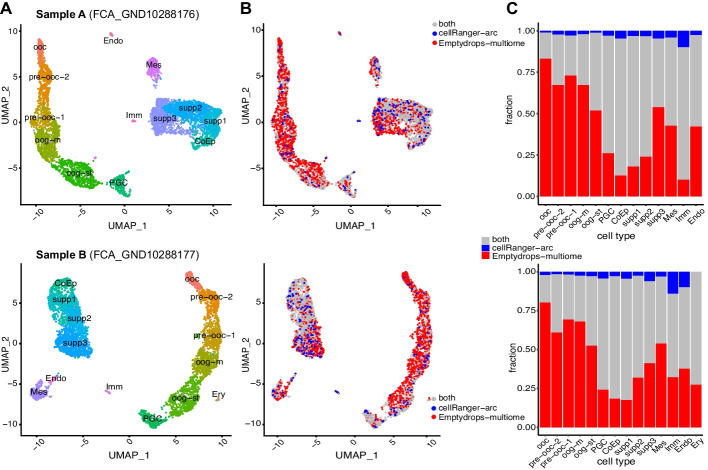
Biological comparison of EmptyDropsMultiome with CellRanger-arc on sample A (top) and sample B (bottom). **A** Uniform Manifold Approximation and Projection (UMAP) of the snRNA-seq data labeled by cell type. We clearly see the main two trajectories for germ cell and somatic cell differentiations and some other small clusters related to immune or erythroid cells. **B** UMAP of the snRNA-seq data where nuclei are colored in gray if they were called by both CellRanger-arc and EmptyDropsMultiome, blue if they were called by CellRanger-arc but not EmptyDropsMultiome, and red if they were called by EmptyDropsMultiome but not CellRanger-arc. CellRanger-arc is prone to missing nuclei in the germ cell differentiation trajectory due to the chromatin being more actively remodeled than in somatic cells [20]. **C** Bar plots showing the fraction of nuclei per cell type called by CellRanger-arc, EmptyDropsMultiome, or both. Oocytes are essentially missed by CellRanger-arc, which recovers only 17% of the ones EmptyDropsMultiome does. Abbreviations: ooc, oocytes; pre-ooc-2, late pre-oocytes; pre-ooc-1, early pre-oocytes; oog-m, oogonia-meiotic; oog-st, oogonia STRA8; PGC, primordial germ cells; CoEp, coelomic epithelium; supp1/2/3, early/middle/late supporting cells; Mes, mesenchymal; Imm, immune; Endo, endothelial; Ery, erythroid

To explore the characteristics of the additional set of nuclei identified by EmptyDropsMultiome, we performed clustering and cell type annotation using the union of droplets called as containing nuclei by either EmptyDropsMultiome or CellRanger-arc and visualize the output using UMAPs (Uniform Manifold Approximation and Projection) [19] (Fig. 4). UMAPs generated using the RNA modality (Fig. 4A, B) show a clear separation between germ cells (right hand side) and a more homogeneous collection of somatic cells (left hand side; Fig. 4A, B; the “Methods” section). When contrasting the sets of nuclei identified by CellRanger-arc and EmptyDropsMultiome, we noted that while both methods well-captured nuclei associated with a somatic cell identity (Fig. 4C), EmptyDropsMultiome identified a much larger fraction of cells associated with a germ cell identity (98.5% of germ cells found by either method were captured by EmptyDropsMultiome compared to 18.5% by CellRanger-arc). This difference was observed in both sample A and in sample B, suggesting that CellRanger-arc is systematically failing to characterize this population of cells (see the “Discussion” section).

To explore the impact of using only the RNA modality for cell calling, we applied EmptyDrops on the two samples, which resulted in the identification of many more putatively nuclei-containing droplets than application of EmptyDropsMultiome (12,932 vs 5056 in sample A and 10,468 vs 5246 in sample B). Among these nuclei-containing droplets, we also observed those corresponding to germ cells that were omitted by CellRanger-arc. However, when focusing on droplets identified exclusively by EmptyDrops, we observed that they were strongly enriched for a high mitochondrial contamination (~ 90% of them reside in the clusters with the highest mitochondrial contamination; see Additional file 1: Tables S1-S2 and Fig. S7). Consequently, we speculate that many of these newly added cells likely correspond to false positives.

In conclusion, we observed that EmptyDropsMultiome strikes a good balance between being able to identify rare populations that are missed by CellRanger-arc (the oocytes) while avoiding calling of large numbers of false positives (the mitochondrially contaminated additional cells found by application of EmptyDrops). Consequently, in conjunction with the simulations, we infer that EmptyDropsMultiome provides a high-performant alternative to existing approaches for calling nuclei-containing droplets from single-cell multiome datasets.

## Discussion

Multiome ATAC + GEX single-nucleus droplet-based assays are being widely adopted in biology, and their increased use necessitates new computational methods to handle various tasks, including the removal of empty droplets. Currently, to our knowledge, there is only one method for removing empty droplets, CellRanger-arc. However, as illustrated above, CellRanger-arc makes parsimonious assumptions about the homogeneity of the cells in the sample, both in terms of their library size and their distribution of fragments in peaks. This can lead it to miss specific cell types in a sample, as was observed in the case of oocytes during gonadal development. Oocytes have highly dynamic chromatin [20, 21], which can result in chromatin becoming accessible in unexpected regions of the genome. As a consequence, oocytes can exhibit a different FRiP distribution compared to other cell types. In fact, 10x Genomics is aware of this problem in the context of immune cells, where granulocytes also have very broadly accessible chromatin and are prone to be missed [18].

An alternative approach to using the FRiP quality control for cell calling would be to first cluster the droplets and then apply different cut-offs in each cluster (typically 3 MAD of the cluster away from the median of the cluster). However, clustering can be ambiguous, especially with large numbers of droplets, making a clustering-free method more desirable for cell calling.

EmptyDropsMultiome is a clustering-free cell-calling algorithm that makes minimal assumptions about the expected types of sought-after nuclei. It relies on modeling the noise (i.e., the soup), which leads to a *p*-value for the RNA and ATAC of each droplet that does not depend on its cell type. One drawback with calling cells based on deviance from the background is that it can lead to the inclusion of damaged cells whose expression and chromatin accessibility profiles deviate from the background. To overcome this challenge, it is necessary—for all methods—to apply post hoc filtering to remove low quality putative cells.

## Conclusions

In conclusion, we show with real data and simulations that CellRanger-arc—the only method currently available for cell/nuclei calling in droplet-based multiome (ATAC + GEX) datasets—can miss specific cell types due to the homogeneity assumptions it uses. In contrast, our proposed method, EmptyDropsMultiome, can detect these and the other cell types, while outperforming EmptyDrops both in real data and in simulations. We envision EmptyDropsMultiome being beneficial to the wider single-cell community, enabling better utilization of multiome data and thus avoiding potential errors in downstream analysis.

## Methods

### Methods development section

#### Defining the ambient RNA and ATAC profiles

In a typical 10x multiome experiment, the majority of barcodes are associated with roughly a single RNA count [13]. We observe this empirically for the samples introduced earlier (Additional file 1: Fig. S9A & C; ~ 500,000 out of more than 736,000 barcodes are associated with ~ 1 RNA count, representing the leftmost peak/cluster of the distribution). Since each 10x multiome kit contains around 80–100,000 beads, the majority of these singleton barcode observations must arise from experimental or computational errors during sample processing, such as the formation of chimeric molecules and sequencing errors that are not properly corrected [13] during initial processing.

The second cluster of the distribution of RNA counts (Additional file 1: Fig. S9A & C) has a mode of around 90 unique reads associated with a barcode. This is unlikely to arise from the formation of chimeric reads or random sequencing errors, which are expected to be rare events. Nevertheless, the overwhelming majority of barcodes in this cluster cannot be associated with droplets containing a nucleus because no more than 16,000 nuclei (as per 10x recommendations) are loaded and ~ 60,000 barcodes contribute to this second cluster. Moreover, true cells are likely to contain on the order of thousands of mRNA molecules. Consequently, we refer to this cluster as the ambient cluster, since it likely consists of cDNA fragments present in the buffer that have been encapsulated by droplets that are nuclei-free. A similar pattern was observed for the ATAC reads (Additional file 1: Fig. S9B & D), with the exception that the ambient cluster and the cluster arising from technical errors are much closer than in the RNA histogram.

#### Creating RNA and ATAC profiles of the soup

Before creating RNA and ATAC profiles of the soup, we wanted to exclude reads that likely arise from technical errors (i.e., reads associated with a barcode that is seen ~ 1 time in the entire dataset) because they can potentially decrease the discriminatory power of our method. To do this, we fit a mixture of three Gaussians (one for the technical-error cluster, another for the ambient cluster, and a third for the real population of nuclei) to the distribution of RNA counts (similarly for ATAC reads) and then only use the reads from the ambient cluster to create the RNA and ATAC soup profiles.

We use ${b}_{A} ({b}_{R})$ and ${l}_{A} ({l}_{R})$ to denote the end of the technical-error ATAC (RNA) cluster and the end of the ambient ATAC (RNA) cluster, respectively. The end of the technical-error cluster is defined as the library size where the probability that a droplet consists of technical-error reads is equal to the probability that it consists of soup. The end of the ambient cluster is chosen as 1.5 sigmas away from its mean for RNA and 2 sigmas for ATAC; we observed that this choice of parameters performs well across several different samples (data not shown).

Following calculation of the above parameters, we can now define the ambient RNA and ATAC distributions as follows. Specifically, let ${t}_{A}^{(c)}, {t}_{R}^{(c)}$ be the counts of droplet c for RNA and ATAC, respectively. We use ${{{X}^{(A)}}_{r,c}}$ to denote the ATAC count (region-by-cell) matrix and ${{{X}^{(R)}}_{g,c}}$ for the RNA count (gene-by-cell) matrix. We calculate the RNA and ATAC profiles of the soup as

$$\begin{array}{l}{am{b}^{(R)}}_{g}= {\sum }_{c: {b}_{R}\le {t}_{R}^{(c)}\le {l}_{R} }{{X}^{(R)}}_{g,c },\\ {am{b}^{(A)}}_{r}= {\sum }_{c: {b}_{A}\le {t}_{A}^{(c)}\le {l}_{A} }{{X}^{(A)}}_{r,c}.\end{array}$$

Finally, we apply the Good-Turing algorithm [22] separately on each of ${\varvec{a}}{\varvec{m}}{{\varvec{b}}}^{({\varvec{A}})}$ and ${\varvec{a}}{\varvec{m}}{{\varvec{b}}}^{({\varvec{R}})}$ to obtain the posterior expectations ${{\underline{p}}^{(A)}}_{r}$ and ${{\underline{p}}^{(R)}}_{g}$ of the proportion of counts assigned to each feature (region or gene) in the soup.

### Testing deviations from the ambient profile

Having computed the ambient RNA and ATAC profiles, we test every droplet (excluding those in the technical-error cluster) for statistical deviations from each of these profiles. The formalism for how to perform this statistical comparison was developed in [12], and we review it briefly here in the context of sc-multiome data.

Our null hypothesis is that all RNA and ATAC counts in a droplet are due to fragments from the soup. The statistical modeling of the random process that generates the counts in a droplet has two parts: the capturing of the free-floating fragments of the soup into the droplet, which is modeled using a Dirichlet distribution, and the sequencing of a sampling of them, which is modeled using the multinomial distribution. Compounding the two, the counts in a droplet under the null hypothesis follow a Dirichlet-multinomial distribution with parameters that are a function of ${{\underline{p}}^{(A)}}_{r}$ or ${{\underline{p}}^{(R)}}_{g}$ in each case.

Given the counts ${{{X}^{(R)}}_{g,c}}$ for a droplet c, and the counts in the ambient profile ${{\underline{p}}^{(R)}}_{g}$, we can compute the likelihood as the probability of the former given the latter,

$$\begin{aligned}{{{{L(\underline p}^{(R)}}_g\vert X}^{(R)}}_{g,c})=\frac{t^{(c)}_R!\Gamma(\alpha)}{\Gamma(t^{(c)}_R+\alpha)}\prod\nolimits_{g=1}^N\quad\frac{\Gamma({X^{(R)}}_{g,c}+\alpha{\underline p^{(R)}}_g)}{{X^{(R)}}_{g,c}!\Gamma(\alpha{\underline p^{(R)}}_g)},\end{aligned}$$

where $N$ is the number of genes and $\alpha$ is used to model overdispersion in the data and is set equal to its maximum likelihood estimate.

Similarly for the ATAC modality,

$$\begin{aligned}{{{{L(\underline{p}}^{(A)}}_{r}|X}^{(A)}}_{r,c})=\frac{t^{(c)}_A! \Gamma (\alpha )}{\Gamma (t^{(c)}_A+\alpha )}{\prod }_{r=1}^{N{\prime}}\quad\frac{\Gamma ({{X}^{(A)}}_{r,c} + \alpha {{\underline{p}}^{(A)}}_{r})}{{{X}^{(A)}}_{r,c}! \Gamma ( \alpha {{\underline{p}}^{(A)}}_{r})},\end{aligned}$$

where $N^\prime$ is the number of regions/called peaks.

We now estimate the RNA *p*-value for droplets c with ${t}_{R}^{(c)}$>${b}_{R}$ using a Monte Carlo simulation with ${M}^{(R)}$ iterations. For each iteration, we sample from a Dirichlet-multinomial distribution with parameters ${{\underline{p}}^{(R)}}_{g}$ and ${t}_{R}^{(c)}$ and then compute the likelihood $L^\prime$ for the resulting RNA count vector. The RNA *p*-value is ${P}_{c}^{(R)}={\frac{{M}_{c}^{(R)}+1}{{M}^{(R)}+1}}$, where ${{{M}_{c}}^{(R)}}$ is the number of iterations for which $L^\prime\le {{{{L(\underline{p}}^{(R)}}_{g}|X}^{(R)}}_{g,c})$ [23]. The ATAC *p*-value for droplets c with ${t}_{A}^{(c)}$>${b}_{A}$ is calculated with another set of ${M}^{(A)}$ iterations as ${P}_{c}^{(A)}={\frac{{M}_{c}^{(A)}+1}{{M}^{(A)}+1}}$.

#### Modeling differences between RNA and ATAC

Since our null hypothesis is that a droplet is empty, we are only concerned with modeling the capturing and sequencing of the ambient counts, not of real nuclei. This is common in both RNA and ATAC—even if distributions of RNA and ATAC counts in real nuclei are vastly different. Consequently, this framework is applicable to both RNA and ATAC, and potentially other epigenetic assays. Of note, since chromatin accessibility in real nuclei is binary and ambient ATAC is continuous, the risk of classifying a nucleus-containing droplet as empty is smaller than in RNA where a cell might happen to look like the soup (especially if the underlying population of cells is homogeneous). On the other hand, the smaller dynamic range for ATAC-seq data might mean that a larger number of iterations is needed to accurately estimate the *p*-values.

#### Aggregating the *p*-values and correcting for multiple testing across barcodes

The two *p*-values are aggregated into one *p*-value using the arithmetic mean, which is known to control for dependence among the *p*-values [24]. The aggregated *p*-values are then corrected using the Benjamini-Hochberg method [25] to control for the false discovery rate (FDR). In the following, we use a FDR threshold of 0.1%.

#### FDR adjustment for soup droplets and optional FDR adjustment for homogeneous samples

To avoid excluding nuclei with a large number of counts that have transcriptomic or chromatin accessibility profiles that are similar to the ambient distribution (a problem for highly homogeneous populations of cells), we apply a k-means classifier (*k* = 2) in the space of log-transformed RNA and ATAC counts. Then, we draw two lines parallel to the k-means line: (1) that passes through the point $( lo{g}_{10}( {l}_{A}), lo{g}_{10}({l}_{R}) )$ and (2) that is ${}^{2}\!\left/ \!{}_{3}\right.$ of the way from the k-means line to the top percentile of the distribution. The FDR for droplets above the second line can optionally be set to 0, to prevent the exclusion of real nuclei that happen to look like the ambient profile, and the FDR for droplets below the first line is set to 1. In the analysis of the simulations and the real data, we implemented EmptyDropsMultiome at FDR ≤ 0.001 without setting FDR to 0 for very large droplets.

### Analysis section: processing, quality control and annotating detected nuclei in real datasets and simulations

#### Simulations

In simulations, where a customizable version of CellRanger-arc was needed (for instance to create the ROC curve), we implemented our own version of the CellRanger-arc algorithm in R. We used this R implementation to call cells on the 10 k PBMC dataset. We then considered as our universe of droplets (i) all the droplets classified by CellRanger-arc as not containing nuclei, (ii) 5000 or 2000 droplets classified as nuclei containing, (iii) 2000 droplets (either randomly sampled from CellRanger-arc identified cells or selected to be monocytes) that we downsampled to 20% RNA and 20% ATAC, and (iv) simulated empty droplets which were generated by multiplying the count vectors of droplets in the ambient peaks by random integers uniformly sampled from [2, 10] for RNA and [2, 40] for ATAC, in order to create a continuum of counts from empty droplets to nuclei-containing droplets.

For the simulations of very small cells, we sampled 4000 CellRanger-arc-identified real cells and then we downsampled 2000 of these cells to 6% of their RNA library size and 2% of their ATAC size.

Moreover, for all comparisons to EmptyDrops, we used the RNA profile of the soup generated from the droplets in the ambient cluster in the RNA space.

#### Real data

On real datasets, we used the following outputs of CellRanger-arc: unfiltered matrix, filtered matrix, metadata file (see data availability). We applied EmptyDropsMultiome on the unfiltered count matrices to ensure that information from all droplets was used. For all comparisons to CellRanger-arc on real data (such as Figs. 3 and 4), we used the barcodes listed in the column names of the filtered count matrix. Finally, for the FRiP values, we used the respective column from the metadata file.

#### EmptyDropsMultiome vs CellRanger-arc on real data

From the set of droplets called by CellRanger-arc or by EmptyDropsMultiome, we selected droplets with mitochondrial (MT-) fraction lower than 3 MAD above the median and with FRiP more than 1 MAD larger than the median. These droplets were then processed with the Seurat package as follows: normalization with SCTransform [26], PCA inference with RunPCA on VariableFeatures, FindNeighbors using the first 50 PCs, then FindClusters on the RNA modality using resolution of 2, and finally RunUMAP using 50 PCs.

We then further removed droplets classified as doublets by scDblFinder [27] with default parameters and the cluster indicated by scDblFinder as containing mostly doublets.

We then performed sample specific quality control.

For sample A, we additionally removed three clusters with the highest mitochondrial fraction (a cluster characterized by high expression of ribosomal genes, a cluster with very clear ZP3 expression, and a cluster with some oogonia-meiotic markers) and one more cluster dominated by EmptyDropsMultiome droplets with a mixture of gene markers.

For sample B, we removed the two clusters with the highest mitochondrial fraction.

We then processed these high-quality droplets with the Seurat package as follows: normalization with SCTransform, PCA inference with RunPCA on VariableFeatures, FindNeighbors using the first 50 PCs, then FindClusters on the RNA modality using a resolution of 1, and finally RunUMAP using 50 PCs. The annotation of the inferred clusters was performed by reproducing the dot plots in [9] (see Additional file 1: Fig. S2).

#### EmptyDropsMultiome vs EmptyDrops on real data

For comparisons to EmptyDrops, we used the *FDR_RNA* column in EmptyDropsMultiome’s output and thresholded at FDR_RNA ≤ 0.1%. This means that the EmptyDrops approach was applied using only the ambient cluster of droplets to profile the soup. Then, for consistency, we implemented the exact same quality control filters described above. Namely, we selected droplets with mitochondrial (MT-) fraction lower than 3 MAD above the median, and with FRiP more than 1 MAD larger than the median, and not classified as doublets by scDblFinder with its default settings. Since the point of the comparison to EmptyDrops is to illustrate how hard downstream analysis becomes due to the large number of extra clusters with EmptyDrops exclusive droplets, we did not implement cluster specific removal. Moreover, almost all of the droplets found exclusively by EmptyDrops are in the top clusters by mitochondrial contamination (Additional file 1: Fig. S7 and Tables S1 and S2) and should hence be removed.

## Supplementary Information

**Additional file 1.** All supplementary figures and tables mentioned in the text.

**Additional file 2.** Review history.

## Data Availability

The dataset (sample A: FCA_GND10287600/FCA_GND10288176 and sample B: FCA_GND10287601/FCA_GND10288177) was obtained from the authors [9] and is available at [10]. EmptyDropsMultiome is available as an R package under the GPL-3.0 license at [28, 29], and the code for the analysis of the data presented in this paper can be found at [30, 31].
