# Supplementary material for: EmptyDropsMultiome discriminates real cells from background in single-cell multiomics assays
Source: Genome Biol. 2024 May 13;25:121. doi: 10.1186/s13059-024-03259-x (PMC11520057; doi:10.1186/s13059-024-03259-x)
Supplement: Supplementary file 1 — Additional file 1. All supplementary figures and tables mentioned in the text. [file 13059_2024_3259_MOESM1_ESM.pdf]

S.1

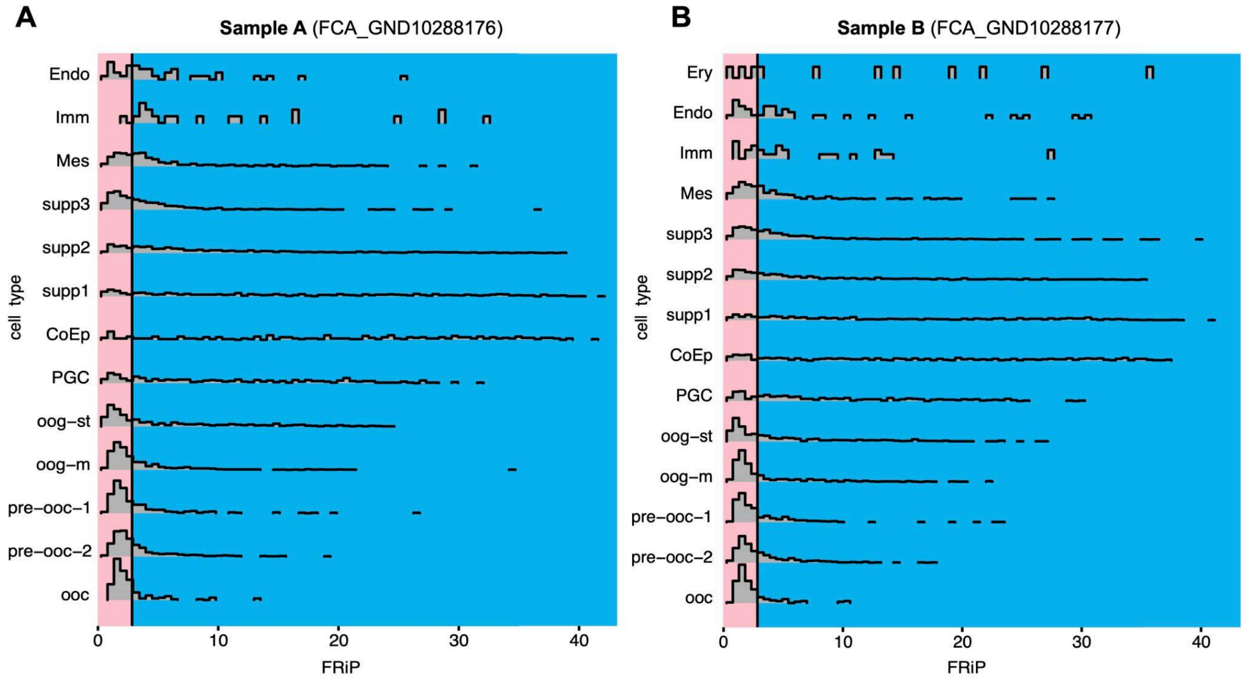

Figure S1. Distribution of fraction of reads in peaks (FRiP) per cell type found by CellRanger-arc or EmptyDropsMultiome for sample A (panel A) and sample B (panel B). Due to the highly active remodeling of the chromatin, germ cells [21] have accessible chromatin at unexpected places, which reduces their FRiP. In pink we see the area rejected by CellRanger-arc.

S.2

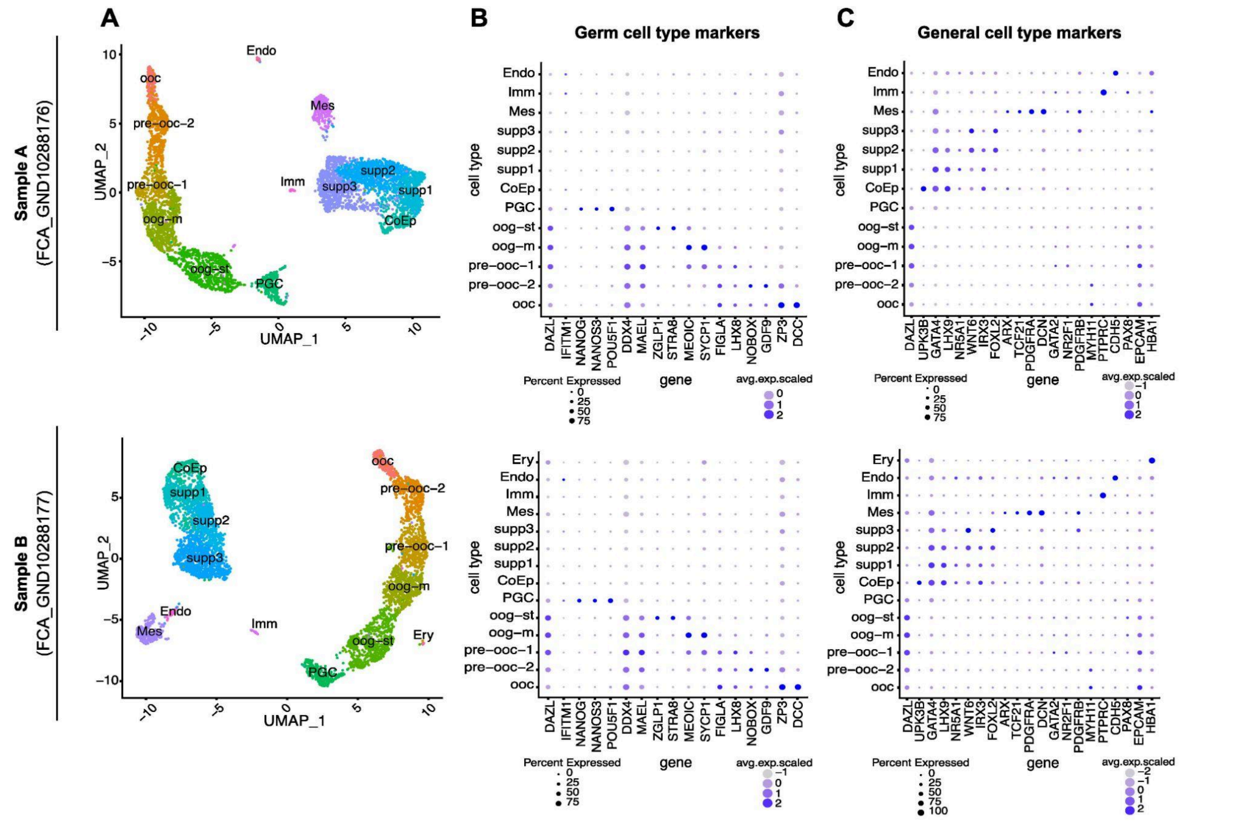

Figure S2. Characterisation of cell types in sample A and B. The differentiation process of germ cells and supporting cells is reflected in the sequence and gradient of gene markers taken from [9]. UMAPs of snRNA-seq data shown in panel **A** are reproduced here from Figure 4 for easier illustration. **B)** Dotplot showcasing the germ cell type markers [9] in each of the identified clusters for sample A (top) and sample B (bottom). **C)** Dotplot showcasing the supporting cell type markers in each of the identified clusters for sample A (top) and sample B (bottom). Dictionary. ooc: oocytes, pre-ooc-2: late pre-oocytes, pre-ooc-1: early pre-oocytes, oog-m: oogonia-meiotic, oog-st:oogonia STRA8, PGC: primordial germ cells, CoEp: coelomic epithelium, supp1/2/3: early/middle/late supporting cells, Mes: mesenchymal, Imm: immune, Endo: endothelial, Ery: Erythroid.

S.3

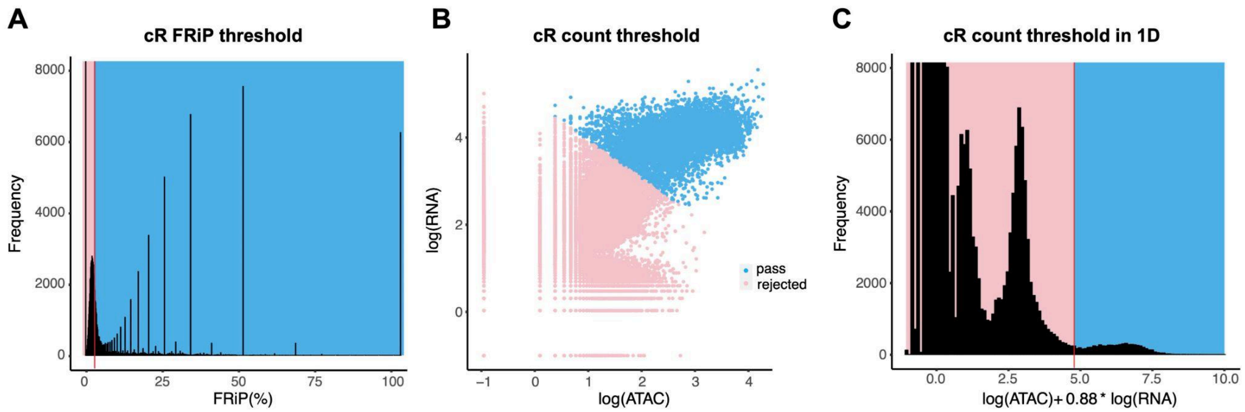

Figure S3. Evaluating the performance of CellRanger-arc on sample B. A) Histogram of the Fraction of Reads in Peaks (FRiP) of all droplets for sample A. Nuclei containing droplets are located within the bell curve and in the right tail of the distribution. The cellRanger-arc threshold (vertical red line) rejects more than half of the nuclei within the bell curve. **B)** CellRanger-arc count threshold. A scatter plot shows the number of log(ATAC) reads (x-axis) versus the unique log(RNA) reads (y-axis) for each droplet, and we observe a continuum in the distribution of counts. Nuclei that pass CellRanger-arc's count threshold are plotted in blue, and those that fail this threshold are plotted in pink. **C)** Histogram of a linear combination of logarithmic counts that is constant along the k-means line. We see a continuum at the location of the threshold (vertical red line).

S.4

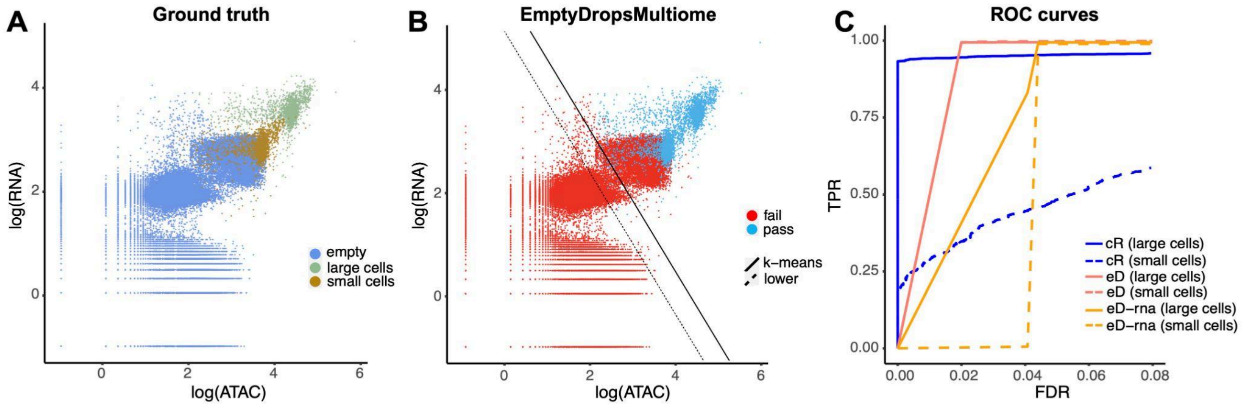

Figure S4. Simulation with 2000 cells of a new cell type with small library size. A) Scatterplot showing the total number of RNA and ATAC counts for the 2000/2000 simulated datasets with one new cell type. Ground truth of the simulated dataset: all the empty droplets from the PBMC dataset and additional simulated empty droplets, 2000 nuclei containing droplets, 2000 small simulated nuclei by subsampling RNA+ATAC and then scrambling the genomic profile of monocytes. **B)** Result of applying EmptyDropsMultiome at FDR=0.1% on the 2000/2000 simulation. The solid line shown is the k-means line used by CellRanger-arc. **C)** ROC curve comparison on the 2000/2000 simulation of EmptyDropsMultiome against a customizable version of CellRanger-arc where we change the intercept of the k-means line (while maintaining its slope). EmptyDropsMultiome finds essentially all the nuclei while maintaining a very low observed FDR, outperforming both CellRanger-arc and EmptyDrops.

S.5

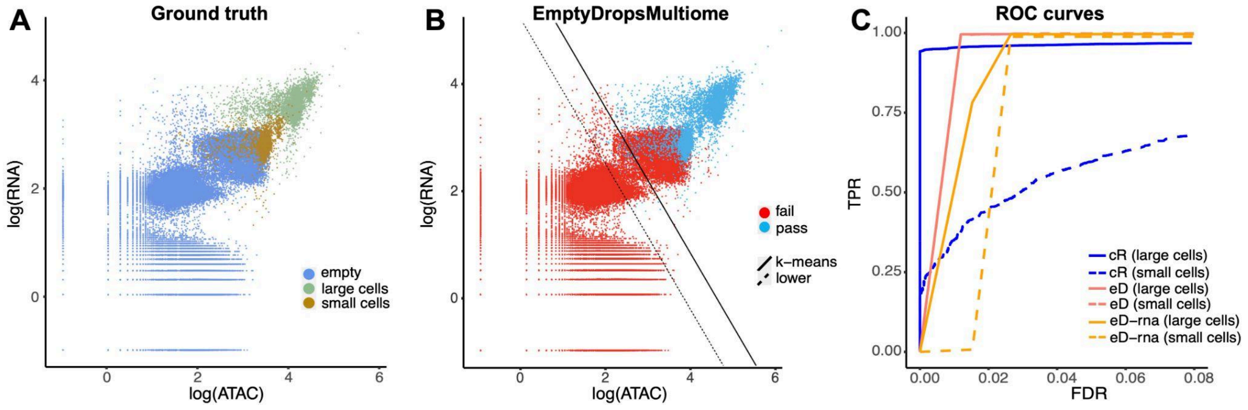

Figure S5. Simulation with 2000 cells of many new cell types of small library size. A) Scatterplot showing the total number of RNA and ATAC counts for one of the 5000/2000 simulated datasets. Ground truth of the simulated dataset: all the empty droplets from the PBMC dataset and additional simulated empty droplets, 5000 nuclei containing droplets, 2000 small simulated nuclei by subsampling RNA+ATAC and then scrambling the genomic profile of any CellRanger-arc identified cell. **B)** Result of applying EmptyDropsMultiome at FDR=0.1% on the 5000/2000 simulation. The solid line shown is the k-means line used by CellRanger-arc. **C)** ROC curve comparison on the 5000/2000 simulation of EmptyDropsMultiome against a customizable version of CellRanger-arc where we change the intercept of the k-means line (while maintaining its slope). EmptyDropsMultiome finds essentially all the nuclei while maintaining a very low observed FDR, outperforming both CellRanger-arc and EmptyDrops.

S.6

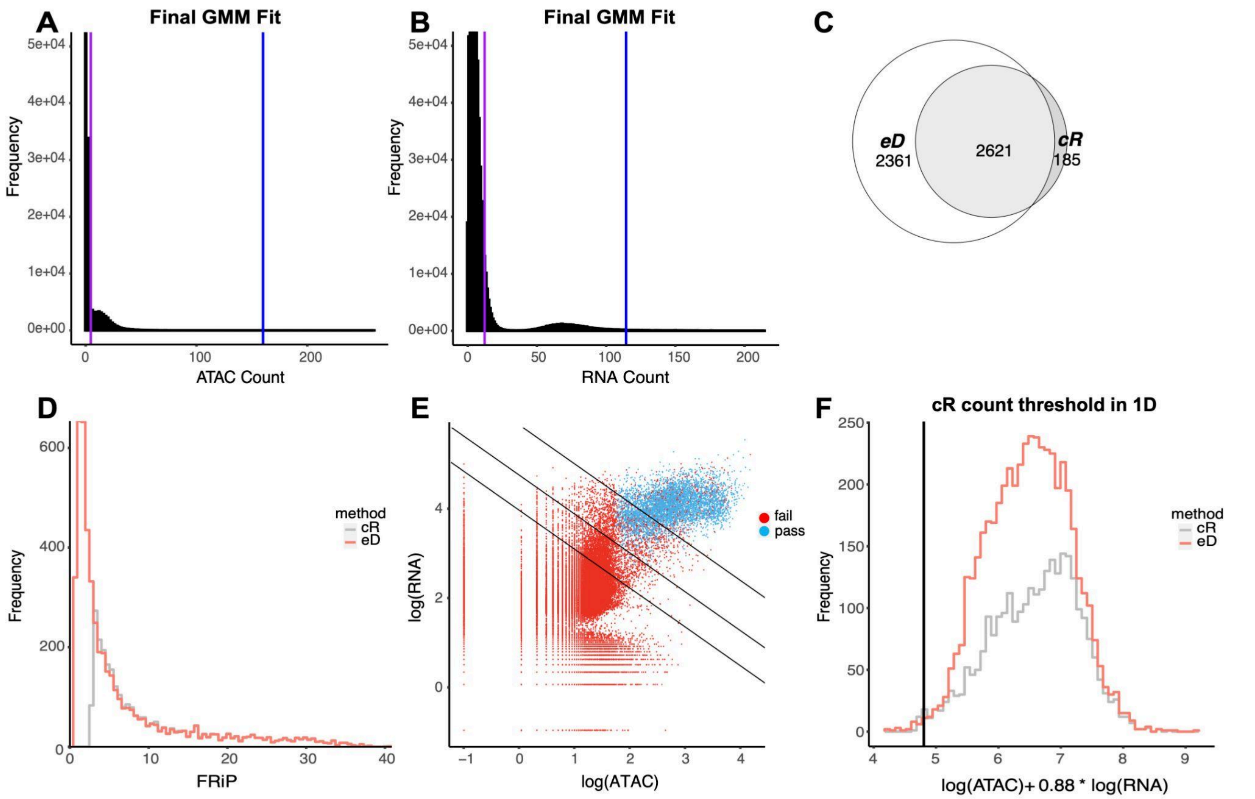

Figure S6. Results of the application of EmptyDropsMultiome on sample B after quality control. A-B) Histograms of ATAC and RNA counts of all the droplets. We profile the soup using only the ambient cluster in the RNA and ATAC. The lower and upper bound of the ambient peak are marked by the purple and blue line and are deduced via a Gaussian Mixtures Model. **C)** Venn diagram showcasing that the droplets selected by Cellranger-arc (cR) are almost a subset of those selected by EmptyDropsMultiome (eD). **D)** Histogram of the FRiP for all droplets selected by CellRager-arc or EmptyDropsMultiome after quality controls. **E)** The distribution of droplets selected by EmptyDropsMultiome in the space of logarithmic counts. **F)** The histogram of the linear combination of logarithmic ATAC and RNA counts that is constant along the k-means line.

S.7

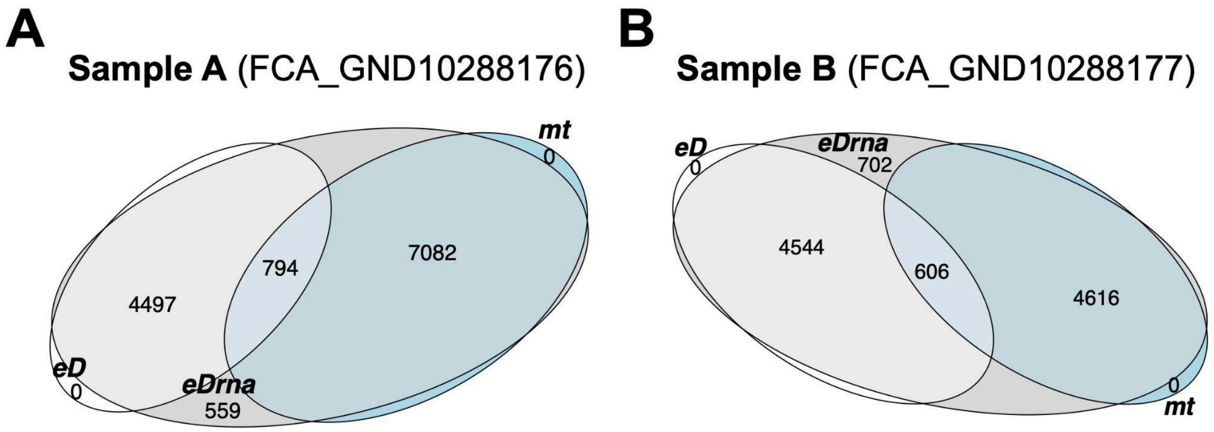

Figure S7. Comparison of EmptyDropsMultiome and EmptyDrops on samples A and B. Venn diagrams showing the nuclei called by EmptyDrops (eDrna) or EmptyDropsMultiome (eD). Although EmptyDrops finds several thousand more cells than EmptyDropsMultiome they are likely false positives since they virtually all reside in the clusters with the largest median mitochondrial contamination (mt).

S.8

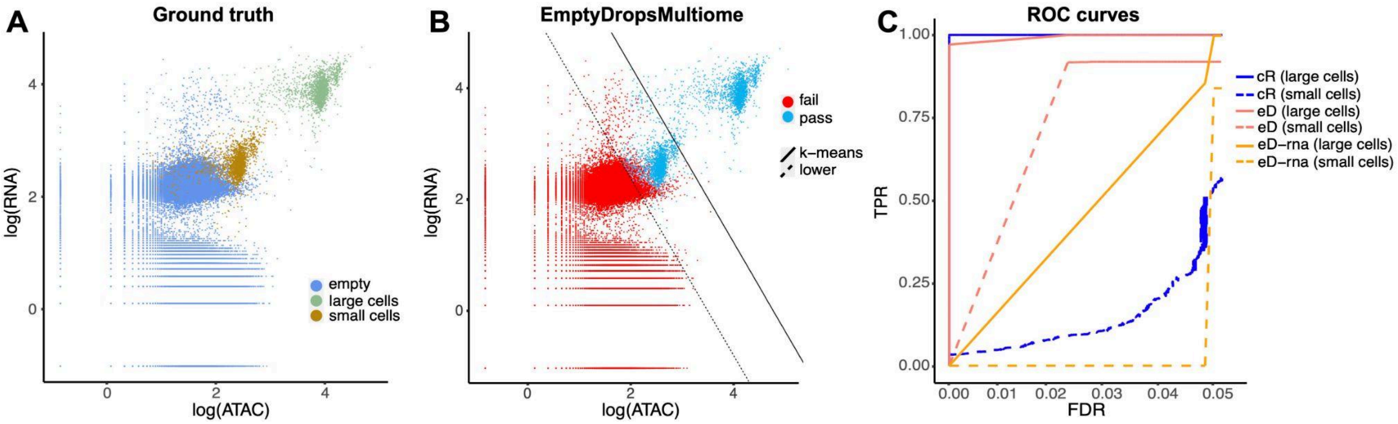

Figure S8. Simulation with 2000 cells of many new cell types with very small library size. The 2000 simulated cells were created by downsampling RNA of real cells to 6% and their ATAC to 2%. Venn diagrams showing the nuclei called by EmptyDrops (eDrna) or EmptyDropsMultiome (eD).

S.9

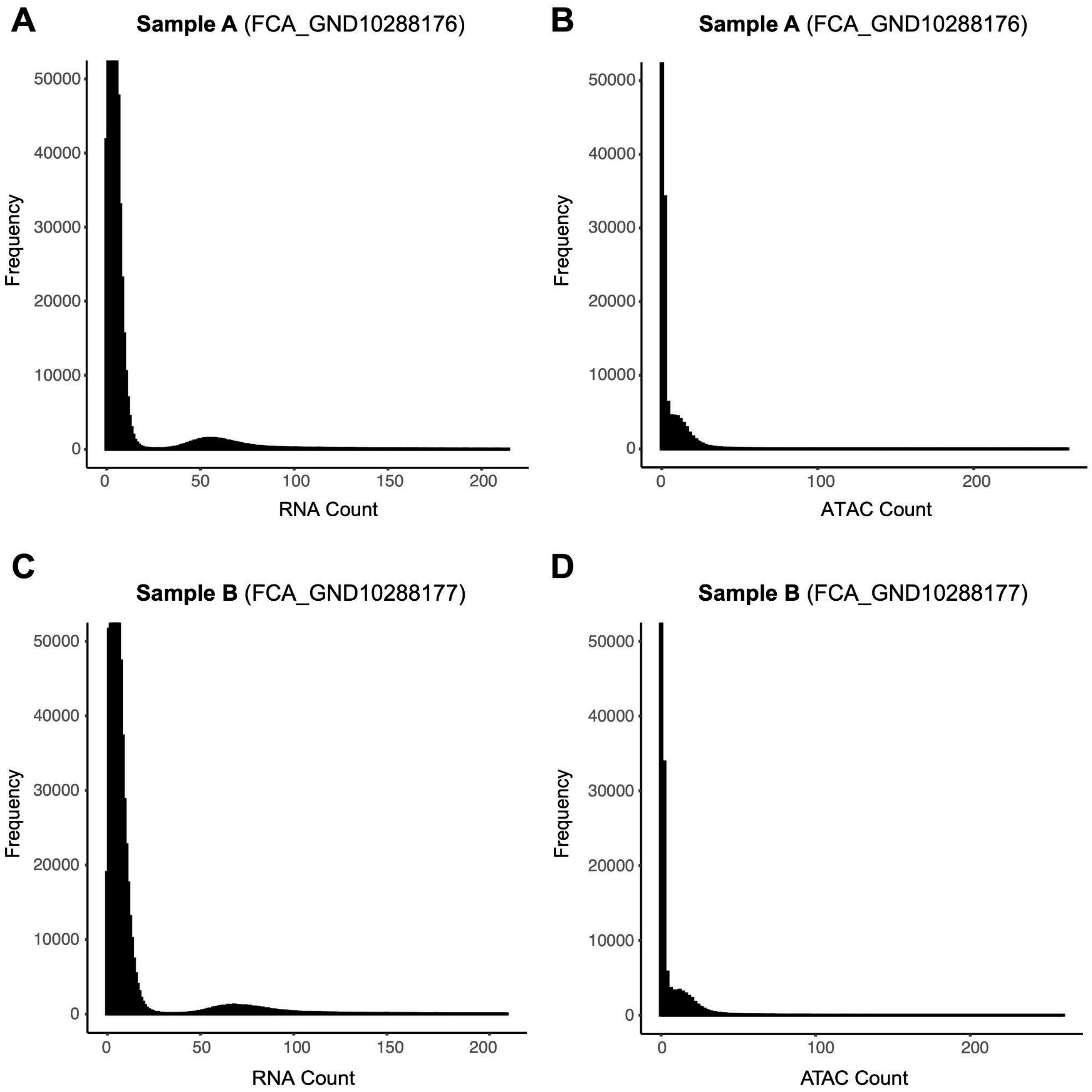

Figure S9. Phenomenology of RNA and ATAC counts. A) Histogram of RNA counts for sample A with the ambient cluster (right peak) clearly separated from the technical error cluster (leftmost peak). **B)** Histogram of ATAC counts, with the technical error cluster (leftmost peak) and the ambient cluster (right peak) close together. **C,D)** Corresponding diagrams for sample B.

cluster	0	1	2	3	4	5	6	7	8	9	10	11	12	13	14	15	16	17
total cells	1698	1685	1468	1272	1238	1012	901	547	531	516	442	423	336	324	309	132	80	26
# of eD cells	84	74	70	1161	52	868	812	309	491	24	401	61	314	232	128	122	73	23
# of rna cells	1697	1685	1468	1272	1238	1012	901	544	531	516	442	423	336	324	305	132	80	26
# of common cells	83	74	70	1161	52	868	812	306	491	24	401	61	314	232	124	122	73	23
% of eD cells	0	0	0	0.9	0	0.9	0.9	0.6	0.9	0	0.9	0.1	0.9	0.7	0.4	0.9	0.9	0.9
% of rna cells	1	1	1	1	1	1	1	1	1	1	1	1	1	1	1	1	1	1
% of common cells	0	0	0	0.9	0	0.9	0.9	0.6	0.9	0	0.9	0.1	0.9	0.7	0.4	0.9	0.9	0.9
median mito %	5.1	4.6	3.4	1.3	3.6	1.6	1	3.4	1.3	3.6	0.9	4.6	0.6	1.1	2.7	0.6	1.1	1.3

Table 1. Comparison of EmptyDropsMultiome and EmptyDrops on sample A. Although EmptyDrops finds several thousand more cells they are likely false positives since they virtually all reside in clusters with large median mitochondrial contamination.

cluster	0	1	2	3	4	5	6	7	8	9	10	11	12	13	14	15	16	17	18
total cells	1289	1205	1084	1076	985	844	613	516	444	435	432	323	272	271	256	191	152	47	33
# of eD cells	1180	69	840	49	99	31	539	459	408	396	265	255	41	243	17	169	35	28	27
# of rna cells	1289	1205	1084	1076	985	844	613	516	444	435	432	323	272	271	256	191	152	47	33
# of common cells	1180	69	840	49	99	31	539	459	408	396	265	255	41	243	17	169	35	28	27
% of eD cells	0.92	0.06	0.77	0.05	0.1	0.04	0.88	0.89	0.92	0.91	0.61	0.79	0.15	0.9	0.07	0.88	0.23	0.6	0.82
% of rna cells	1	1	1	1	1	1	1	1	1	1	1	1	1	1	1	1	1	1	1
% of common cells	0.92	0.06	0.77	0.05	0.1	0.04	0.88	0.89	0.92	0.91	0.61	0.79	0.15	0.9	0.07	0.88	0.23	0.6	0.82
median mito %	1.21	2.45	1.65	2.93	3.16	2.77	1.24	1.05	0.85	0.91	2.87	0.93	3.02	0.35	2.82	0.75	1.92	1.36	1.03

Table 2. Comparison of EmptyDropsMultiome and EmptyDrops on sample B. As in sample A, EmptyDrops finds roughly equal numbers of cells but thousands more droplets which are likely false since they virtually all reside in clusters with large median mitochondrial contamination.

	0	1	2	3	4	5	6	7	8	9	10	11	12	13
**total cells**	863	678	653	627	535	466	310	266	262	209	177	155	38	20
**# of eD cells**	834	647	639	619	518	455	301	255	254	199	175	147	37	18
**# of cR cells**	658	279	214	302	439	153	84	153	194	183	30	106	22	18
**# of common cells**	629	248	200	294	422	142	75	142	186	173	28	98	21	16
**% of eD cells**	0.97	0.95	0.98	0.99	0.97	0.98	0.97	0.96	0.97	0.95	0.99	0.95	0.97	0.9
**% of cR cells**	0.76	0.41	0.33	0.48	0.82	0.33	0.27	0.58	0.74	0.88	**0.17**	0.68	0.58	0.9
**% of common cells**	0.73	0.37	0.31	0.47	0.79	0.3	0.24	0.53	0.71	0.83	0.16	0.63	0.55	0.8

Table 3. Comparison of EmptyDropsMultiome (eD) and CellRanger-arc (cR) on sample A. Cluster 10 is the cluster with the oocytes and CellRanger-arc finds only 17% of these cells.

	0	1	2	3	4	5	6	7	8	9	10	11	12	13	14
**total cells**	901	573	544	540	478	455	409	294	275	267	187	165	40	28	11
**# of eD cells**	845	547	530	526	464	447	402	285	263	259	183	160	36	24	11
**# of cR cells**	531	473	371	173	241	140	160	136	209	218	37	65	25	19	8
**# of common cells**	475	447	357	159	227	132	153	127	197	210	33	60	21	15	8
**% of eD cells**	0.94	0.95	0.97	0.97	0.97	0.98	0.98	0.97	0.96	0.97	0.98	0.97	0.9	0.86	1
**% of cR cells**	0.59	0.83	0.68	0.32	0.5	0.31	0.39	0.46	0.76	0.82	**0.2**	0.39	0.62	0.68	0.73
**% of common cells**	0.53	0.78	0.66	0.29	0.47	0.29	0.37	0.43	0.72	0.79	0.18	0.36	0.52	0.54	0.73

Table 4. Comparison of EmptyDropsMultiome (eD) and CellRanger-arc (cR) on sample B. Cluster 10 is the cluster with the oocytes and CellRanger-arc finds only 20% of these cells.
