# Supplementary material for: EmptyDropsMultiome discriminates real cells from background in single-cell multiomics assays
Source: Genome Biol. 2024 May 13;25:121. doi: 10.1186/s13059-024-03259-x (PMC11520057; doi:10.1186/s13059-024-03259-x)
Supplement: Supplementary file 2 — Additional file 2. Review history. [file 13059_2024_3259_MOESM2_ESM.docx]

Review History

**First round of review**

**Reviewer 1**

**Were you able to assess all statistics in the manuscript, including the appropriateness of statistical tests used?** Yes: appropriate and rigorous.

**Were you able to directly test the methods?** No.

**Comments to author:**

Megas and colleagues develop a new method, EmptyDropsMultiome, which greatly improves the identification and calling of high-quality nuclei in single cell multiome datasets. The authors demonstrate the performance of this method using simulations and real datasets of human gonadal development and clearly show improved accuracy of their method, compared to the CellRanger method that is currently standard in the field. Importantly, EmptyDropsMultiome retrieves a large number of nuclei in real data missed by CellRanger, including certain cell types that are almost completely lost using the CellRanger method. The manuscript is clearly written and the figures are to the point and easy to understand. The methods are detailed, datasets and code publicly available, and the EmptyDropsMultiome package is available on Github. This will be a highly useful and widely used tool for the community, and importantly, will rescue enormous amounts of data that are currently lost for downstream analysis using current methods. I support the rapid publication of this manuscript as is.

**Reviewer 2**

**Were you able to assess all statistics in the manuscript, including the appropriateness of statistical tests used?** Yes. All statistics in the manuscript are solid.

**Were you able to directly test the methods?** No.

**Comments to author:**

Comments to Author:

In this work, Megas et al. address the problem of filtering nuclei-free droplets containing only ambient RNA and chromatin fragments from single-cell multiome data. They establish EmptyDropsMultiome building on the statistical framework of the previous EmptyDrops method for the detection of empty droplets in single-cell/nucleus RNA-seq data.

Since CellRanger-arc supplied by the 10x software pipeline for the analysis of Multiome data is currently the only available method for this task, the proposed method serves an urgent need.

The authors discuss the arbitrary filtering criteria of CellRanger-arc and showcase with simulated and real datasets that this pipeline may discard a large fraction of nuclei-containing droplets.

EmptyDropsMultiome represents a robust statistical method with the benefit of not relying on arbitrary clustering of the data. Droplets are discarded based on their probability to be drawn from an expected count distribution inferred from bona fide empty droplets.

Using simulated data and real datasets the authors demonstrate that EmptyDropsMultiome avoids filtering of cells with low RNA and/or ATAC-seq counts, and they show the additional benefit of modeling the ATAC-seq modality on top of the gene expression as implemented in EmptyDrops.

Since generation of Multiome data by the community has been facilitated by robust protocols such as 10x Multiome, EmptyDropsMultiome represents a very useful method to optimize the processing of such data.

The method and the benchmarking are statistically solid and the manuscript is well written.

However, a number of points should be addressed by the authors prior to publication:

1. It seems that the EmptyDrops-paradigm would be useful for filtering droplets in unimodal single-cell ATAC-seq data. I'm not familiar with the available spectrum of methods for calling empty droplets in scATAC-seq data, but it may be a useful extension to introduce EmptyDropsATAC alongside EmptyDrops and EmptyDropsMultiome.

2. It is not entirely clear to me how the FRiP filtering threshold of CellRanger-arc is determined. The authors showcase an example where they argue that inclusion of spurious peaks entails a high FRiP threshold leading to exclusion of real nuclei. Naïvely, I would expect that inclusion of spurious peaks increases the FRiP for each cell. Apparently, the CellRanger-arc threshold is determined as a function of the FRiP distribution such that a global increase in FRiP leads to a higher threshold. Could the authors comment whether information is available from the CellRanger-arc documentation how exactly the threshold is selected?

3. A mixture of three Gaussians is fit to the RNA (or ATAC) profiles to capture the technical-error cluster, the ambient population of droplets, and the nuclei-containing droplets. I'd expect the counts to rather follow negative binomial distributions (capturing sampling noise and global variability in cell-to-cell RNA/ATAC-seq efficiency). Such distributions rather resemble log-normal distributions. Did the authors perform log transformation of the counts prior to fitting Gaussians? Please show the quality of the fit.

4. In lines 176 and 180, I believe that the arguments on the left hand side have to be switched, since the likelihood of observing counts X given the model parameters p is computed, i.e. L(X|p).

5. How is the number of iterations (M^R or M^A) determined (line 183)? How strongly does the filtering depend on the choice?

6. For the benchmarking, the authors simulate a continuous profile of empty droplets by multiplying counts with random integer numbers. The authors should also include the benchmarking results when using unmodified empty droplets, since it is unclear to what extent the simulated empty droplets resemble real data. Moreover, it would be informative to also include the complementary scenario, where simulations are performed based on the EmptyDropsMultiome calls.

**Authors Response**

**Point-by-point responses to the reviewers’ comments:**

**Reviewer #1**

Megas and colleagues develop a new method, EmptyDropsMultiome, which greatly improves the identification and calling of high-quality nuclei in single cell multiome datasets. The authors demonstrate the performance of this method using simulations and real datasets of human gonadal development and clearly show improved accuracy of their method, compared to the CellRanger method that is currently standard in the field. Importantly, EmptyDropsMultiome retrieves a large number of nuclei in real data missed by CellRanger, including certain cell types that are almost completely lost using the CellRanger method. The manuscript is clearly written and the figures are to the point and easy to understand. The methods are detailed, datasets and code publicly available, and the EmptyDropsMultiome package is available on Github. This will be a highly useful and widely used tool for the community, and importantly, will rescue enormous amounts of data that are currently lost for downstream analysis using current methods. I support the rapid publication of this manuscript as is.

We thank the reviewer for their positive comments on our work.

**Reviewer #2**

*In this work, Megas et al. address the problem of filtering nuclei-free droplets containing only ambient RNA and chromatin fragments from single-cell multiome data. They establish EmptyDropsMultiome building on the statistical framework of the previous EmptyDrops method for the detection of empty droplets in single-cell/nucleus RNA-seq data.*

*Since CellRanger-arc supplied by the 10x software pipeline for the analysis of Multiome data is currently the only available method for this task, the proposed method serves an urgent need.*

*The authors discuss the arbitrary filtering criteria of CellRanger-arc and showcase with simulated and real datasets that this pipeline may discard a large fraction of nuclei-containing droplets.*

*EmptyDropsMultiome represents a robust statistical method with the benefit of not relying on arbitrary clustering of the data. Droplets are discarded based on their probability to be drawn from an expected count distribution inferred from bona fide empty droplets.*

*Using simulated data and real datasets the authors demonstrate that EmptyDropsMultiome avoids filtering of cells with low RNA and/or ATAC-seq counts, and they show the additional benefit of modeling the ATAC-seq modality on top of the gene expression as implemented in EmptyDrops.*

*Since generation of Multiome data by the community has been facilitated by robust protocols such as 10x Multiome, EmptyDropsMultiome represents a very useful method to optimize the processing of such data.*

*The method and the benchmarking are statistically solid and the manuscript is well written.*

We thank the reviewer for their positive comments on our work.

*1. It seems that the EmptyDrops-paradigm would be useful for filtering droplets in unimodal single-cell ATAC-seq data. I'm not familiar with the available spectrum of methods for calling empty droplets in scATAC-seq data, but it may be a useful extension to introduce EmptyDropsATAC alongside EmptyDrops and EmptyDropsMultiome.*

This is indeed a great idea for a follow-up project, but is beyond the scope of what we could accomplish in the short time in which the manuscript has to be revised for resubmission. Moreover, we feel that our focus on nuclei calling for sc-multiome data is justified since, moving forward, it is highly likely that most datasets will be generated using multiome technologies than with scATAC alone. In particular, the combined approach makes cell type annotation much easier and, consequently, the majority of recent papers have focused on using this technology.

*2. It is not entirely clear to me how the FRiP filtering threshold of CellRanger-arc is determined. The authors showcase an example where they argue that inclusion of spurious peaks entails a high FRiP threshold leading to exclusion of real nuclei. Naïvely, I would expect that inclusion of spurious peaks increases the FRiP for each cell. Apparently, the CellRanger-arc threshold is determined as a function of the FRiP distribution such that a global increase in FRiP leads to a higher threshold. Could the authors comment whether information is available from the CellRanger-arc documentation how exactly the threshold is selected?*

The joint cell calling by cellranger-arc is described on this webpage <https://support.10xgenomics.com/single-cell-multiome-atac-gex/software/pipelines/latest/algorithms/cell-calling>. On the first step of the cell calling process by cellranger-arc, barcode i is rejected if FRiP < (% of genome in peaks). This is the reason why including many false peaks can cause a cell to be wrongly rejected since it increases the threshold without substantially increasing its FRiP.

*3. A mixture of three Gaussians is fit to the RNA (or ATAC) profiles to capture the technical-error cluster, the ambient population of droplets, and the nuclei-containing droplets. I'd expect the counts to rather follow negative binomial distributions (capturing sampling noise and global variability in cell-to-cell RNA/ATAC-seq efficiency). Such distributions rather resemble log-normal distributions. Did the authors perform log transformation of the counts prior to fitting Gaussians? Please show the quality of the fit.*

We apologize for the lack of clarity in the figure labels/legends. The x-axes of RNA and ATAC profiles are the total count of reads, also known as the library size of the droplet, not the counts of any individual gene across cells. For this reason we feel that our choice of a mixture of Normal distributions is justified. We elaborate our thinking in the following three points:

1) Several papers have shown the usefulness of Negative Binomial distributions for modeling the counts per gene in real cells in single-cell experiments [see Lopez et al Nature Methods 2018]. However, due to the additive property of Negative Binomial distributions in the rate-dispersion parameterization, this would translate to a negative binomial distribution for the library size only if the dispersion parameter is the same for all genes.
2) Moreover, even if the library size of *true* cells followed a negative binomial distribution, this would not imply that the library size of *empty* droplets (our only concern in creating the noise profile) would also follow it.
3) In addition, we also more formally assessed whether negative binomials provide a better fit for the ambient noise than our mixture of three Normal distributions. After doing this analysis, we concluded that the mixture of normals performed better. More specifically, we used the SIBERG package in R to fit two Negative Binomials for RNA and ATAC (initialized with values to ensure convergence to the technical-error and ambient-noise peaks).

   We compared the Normal Mixture model vs NB mixture model using the Bayesian Information Criterion, a standard metric to compare models that accounts for models’ potentially different number of parameters. For RNA, the BIC of our model is lower (i.e., better) for both samples. For ATAC, the NB mixture model does not converge and we had to expand the search to ZINB distributions to achieve convergence.

| BIC scores | Mixture of 3 Normals | Mixture of 2 (ZI)NB | Better |
| --- | --- | --- | --- |
| sample_8176_RNA | 4676103.52 | 10857598.92 | yes |
| sample_8177_RNA | 5044075.11 | 11588357.43 | yes |
| sample_8176_ATAC | 3184611.58 | 3215119.54 | yes |
| sample_8177_ATAC | 3159587.58 | 3175685.54 | yes |

*4. In lines 176 and 180, I believe that the arguments on the left hand side have to be switched, since the likelihood of observing counts X given the model parameters p is computed, i.e. L(X|p).*

We thank the reviewer for pointing this out. There are two notations for the likelihood: one that explicitly demonstrates its equality to the probability of observing the data given the model parameters, and another notation (opted for in our text and in many other manuscripts) that showcases that the likelihood is a function of the parameters given the data.

We would be happy to change this notation if Genome Biology follows the notational convention suggested by the reviewer.

*5. How is the number of iterations (M^R or M^A) determined (line 183)? How strongly does the filtering depend on the choice?*

This is a great observation and indeed the number of simulations does matter in whether a cell will be correctly classified. For instance if we choose M=100 simulations, then the lowest non-zero p-value that a cell could get is (0+1)/(100+1), so no p-value would pass the default 0.001 threshold.

Ideally the number of iterations should be very large, so we recommend that the user chooses a high number such as 20000 (chosen by default).

To warn the user against the possibility of a barcode being rejected because of small M, we include in the output dataframe two columns, Limited_RNA and Limited_ATAC, which are logical variables, indicating whether a lower p-value could be obtained by increasing the number of iterations.

*6. For the benchmarking, the authors simulate a continuous profile of empty droplets by multiplying counts with random integer numbers. The authors should also include the benchmarking results when using unmodified empty droplets, since it is unclear to what extent the simulated empty droplets resemble real data. Moreover, it would be informative to also include the complementary scenario, where simulations are performed based on the EmptyDropsMultiome calls.*

In our simulation we used a clean dataset which provides a reliable ground truth, and then used it to create a simulation where the signal to noise ratio is low by simulating new and large empty droplets (by multiplying the counts of existing empty droplets). The reviewer brings up the possibility that our way of simulating the noise is incorrect and therefore our simulation does not demonstrate the validity of our method.

To address this point we created a new simulation where we don’t simulate new empty droplets, but instead downsample the existing cell types even more to bring them down to noise levels.

In the new simulations the performance gap with the competing algorithms in fact increases, as shown by the ROC curves, and CellRanger-arc completely misses the newly introduced cells because they lie below the k-means line.

**Second round of review**

**Reviewer 2**

The authors addressed all of my concerns and questions in the revised manuscript, which is now ready for publication.
